# Cardiovascular disease risk in early rheumatoid arthritis: the impact of cartilage oligomeric matrix protein (COMP) and disease activity

**DOI:** 10.1186/s41927-023-00367-2

**Published:** 2023-12-01

**Authors:** Emil Rydell, Lennart TH Jacobsson, Tore Saxne, Carl Turesson

**Affiliations:** 1 Rheumatology, Department of Clinical Sciences, Lund University, Jan Waldenströms gata 1B, Malmö, Malmö, SE-205 02 Sweden; 2 Department of Rheumatology and Inflammation Research, Sahlgrenska Academy at Gothenburg University, Guldhedsgatan 10 A, Göteborg, SE-405 30 Sweden; 3 Rheumatology and Molecular Skeletal Biology, Department of Clinical Sciences, Lund University, Kioskgatan 3, Lund, Lund, SE-222 42 Sweden; 4 Department of Rheumatology, Skåne University Hospital, Jan Waldenströms gata 1B, Malmö, SE-205 02 Sweden

**Keywords:** Arthritis, rheumatoid, Prognosis, Biomarkers, Cardiovascular Diseases.

## Abstract

**Background:**

To investigate whether baseline serum cartilage oligomeric matrix protein (COMP), patient characteristics, traditional cardiovascular disease (CVD) risk factors and disease activity over time predict CVD, in early rheumatoid arthritis (RA).

**Methods:**

This study included patients with early RA (< 12 months disease duration) (n = 233) recruited 1995–2005. Potential predictors of CVD and coronary artery disease (CAD) were assessed using Cox regression.

**Results:**

A first ever diagnosis of CVD occurred in 70 patients, and CAD in 52. Age, sex, hypertension and diabetes predicted CVD and CAD. COMP was associated with increased risk of CVD and CAD [crude hazard ratios (HRs) per SD 1.45; 95% CI 1.17–1.80 and 1.51; 95% CI 1.18–1.92, respectively]. When adjusted for age, sex, hypertension, diabetes and ESR, results where similar but did not reach significance [HRs 1.32, 95% CI 0.99–1.74 and 1.35, 95% CI 0.99–1.86]. Baseline disease activity did not independently predict CVD. High DAS28 (> 5.1) at two years was associated with increased risk of subsequent CVD [adjusted HR 2.58; 95% CI 1.10–6.04] and CAD. ESR and CRP at two years as well as cumulative disease activity over 2 years independently predicted CVD and CAD.

**Conclusion:**

COMP may be a novel predictor of CVD and CAD in RA. Active disease two years after RA diagnosis, as well as cumulative disease activity, was associated with increased risk of CVD and CAD, independent of traditional CVD risk factors. Awareness of the particularly increased CVD risk among difficult to treat patients is important in order to further reduce CVD in RA.

**Supplementary Information:**

The online version contains supplementary material available at 10.1186/s41927-023-00367-2.

## Background

Patients with rheumatoid arthritis (RA) have been shown to have an increased risk of cardiovascular disease (CVD) compared to the general population, with a relative risk between 1.4 and 2.1 [1–4]. The risk has been reported to be increased already in early RA [5]. Traditional CVD risk factors are predictive of CVD in RA [6]. However, the increased risk of CVD in RA has been shown to be partly independent of traditional CVD risk factors [7], indicating additional mechanisms contributing to its development.

Levels of RA disease activity and inflammation have been associated with endothelial dysfunction, subclinical- and clinical atherosclerosis [8, 9]. Markers of inflammation have been shown to be associated with CVD in patients with RA [10–14]. The ability of disease activity score in 28 joints (DAS28) to predict CVD is uncertain when analysed as single measures [15–17], whereas multiple measures over time, indicating cumulative disease activity, may be more useful [10, 13, 16, 18, 19]. Results on the impact of the RA-associated autoantibodies rheumatoid factor (RF) and anti-citrullinated protein antibodies (ACPA) on CVD risk are mixed [10, 13, 17, 20, 21].

Cartilage oligomeric matrix protein (COMP, thrombospondin 5), a marker of cartilage turnover, is normally found in synovial joints [22] but also in arterial walls [23], with greater abundance in atherosclerotic plaques, particularly those demonstrating vulnerable characteristics [24]. In RA, while associations between serum-COMP and progression of joint damage have been demonstrated [25–28], its association with CVD has not been studied.

In the recently updated EULAR recommendations, it is suggested that general population CVD risk algorithms should be adapted for patients with RA with an 1.5 multiplication factor for all patients [29]. This method estimates risk on a group level and does not take into consideration disease associated factors that influence risk among RA patients. There is a need for improved individual prediction of CVD in RA. Hence, the aim of this study was to evaluate how a potential novel prognostic marker such as COMP, as well as disease activity measures, C-reactive protein (CRP) and erythrocyte sedimentation rate (ESR), RA associated auto-antibodies, traditional CVD risk factors, and time to disease-modifying anti-rheumatic drugs (DMARD) treatment and inflammation control over time predict future CVD in patients with early RA.

## Methods

### Patients

An inception cohort of 233 consecutive patients with early RA was investigated, as previously described [30, 31]. Patients were recruited from the rheumatology outpatient clinic of Skåne University Hospital Malmö, the only hospital serving the city, or from the four rheumatologists in private practice in the area, between 1995 and 2005.

The patients were diagnosed with RA by a specialist in rheumatology, fulfilled the 1987 American College of Rheumatology (ACR) classification criteria for RA [32] and had duration of symptoms ≤ 12 months at the time of inclusion. There were no additional exclusion criteria.

### Clinical assessment

Patients were followed according to a structured program with evaluations at baseline, 6 months, 1 year and 2 years. The same rheumatologist (see acknowledgements) performed all the clinical examinations. For each follow-up visit, blood samples were analysed, and disease activity parameters recorded. Disability was assessed using the Swedish version of Health Assessment Questionnaire (HAQ) [33]. All patients were managed according to usual care with no pre-specified protocol for anti-rheumatic treatment. The patients were included before the current practice of treat to target [34] was implemented, and before treatment with biologic DMARDs came into widespread use.

Information on height, weight, and smoking history (current/previous/never) was collected at inclusion by a questionnaire filled out by the patients. The time from symptom onset to start of DMARD treatment, and traditional CVD risk factors at the time of inclusion, were assessed by systematic case record reviews.

The presence of hypertension, diabetes or hyperlipidaemia before RA diagnosis was defined as a corresponding diagnosis in the case records. For hyperlipidaemia, only cases with elevated lipid levels in the case record review were classified as having this exposure in the study.

Data on biologic DMARD treatment during the study period was obtained through a regional biologics register [35].

### Laboratory investigations

IgM RF was analysed using ELISA, which was calibrated against the World Health Organization (WHO) RF reference preparation. Anti-cyclic citrullinated peptide antibodies (anti-CCP) were analysed using the Quanta Lite CCP IgG ELISA (INOVA Diagnostics, US). ESR and CRP were assessed according to standard methods at Malmö University Hospital. Serum COMP concentrations were determined using a sandwich ELISA (AnaMar, Lund, Sweden). The detection limit of the assay is < 0.1U/L, and its intra-assay and inter-assay coefficient is < 5% [26].

### Cardiovascular disease definitions, data sources and outcome variables

Definitions of CVD in this study were based on codes from the 8th, 9th and 10th version of International Classification of Disease (ICD): (Supplementary Table S1). ICD-codes from 1969 to 2019 were retrieved from the Swedish National Hospital Discharge Register and Causes of Death Register. In Sweden, reporting of underlying and contributing causes of death to the Cause of Death Register is mandatory.

In the analyses on potential prognostic markers, the primary outcome was first diagnosis of CVD (coronary artery disease (CAD), cerebrovascular disease or peripheral artery disease) during the follow-up. Secondary outcomes were the first diagnosis of each respective CVD subcategory: CAD, cerebrovascular disease and peripheral artery disease, during the follow-up.

### Statistical analysis

The relation between potential predictors and outcomes of CVD, and of each subcategory, were examined in Cox regression models. Patients were censored at death or at the end of follow-up (December 31, 2019).

Analyses of COMP, sex, age, time to DMARD, traditional CVD risk factors, serological status, DAS28, HAQ, ESR and CRP as potential baseline predictors of CVD and respective subcategories were performed univariately and adjusted for age, sex, hypertension and diabetes (based on results from the univariate models). To assess whether a potential effect of COMP on the risk of CVD and CAD was influenced by inflammation, we also included ESR in the models. Patients with a registered diagnosis of CVD before inclusion were excluded from analyses of CVD and the respective CVD subcategory.

In separate models, analyses of DAS28, HAQ, ESR and CRP at the 2-years follow-up visit and cumulative measures of DAS28, HAQ and ESR during the first two years after inclusion as potential predictors of subsequent CVD and respective subcategories, were performed univariately and adjusted for age, sex, hypertension and diabetes at baseline (based on results from the univariate models of baseline predictors). For the analyses of the subcategory CAD as outcome, diabetes was not included in the multivariate models due to its lack of independent association with this outcome, and the limited number of patients with CAD in these analyses. Patients with a registered diagnosis of CVD before the 2-year follow-up were excluded from analyses of CVD and the respective CVD subcategory.

All cumulative measures were calculated as area under curve (AUC) from data at inclusion, 6 months, 1 year and 2 years. Risk estimates for continuous disease activity measures (including AUC) were analysed and presented as per standard deviation (SD) to facilitate comparison between potential predictors. Current-, previous-, and ever smoking (current or previous) were each compared to the reference category, never smoking. Body mass index (BMI) was included as a continuous variable.

During part of the study period, high-sensitivity CRP analysis was not available and values between 0 and 9 mg/l were reported as < 9 mg/l. In analyses, CRP was therefore included as a dichotomized variable; that is, above versus below the median (9 mg/l) at inclusion and above versus below the 75th percentile (12 mg/l) at 2 years (since the median at 2 year was < 9 mg/l).

In exploratory analyses, the relation between baseline COMP and CVD risk from inclusion through follow-up was investigated in models stratified by cumulative disease activity over 2 years (above vs. below the median of AUC for DAS28). Patients with a registered diagnosis of CVD before inclusion were excluded from these analyses.

For dichotomous variables, the proportional hazards (PH) assumption was tested by visual examination of plots for log-minus-log function and CVD follow-up time. For continuous variables, the PH assumption was tested by correlation of the partial residuals from cox regression analyses with the rank of CVD follow-up time, and r > 0.3 with p < 0.05 was used as the cut off for exclusion. All models fulfilled the PH assumptions.

Statistical analysis was performed using IBM SPSS Statistics version 28.0, Armonk, NY, IBM Corp.

## Results

### Patient characteristics and traditional cardiovascular risk factors

A total of 232 patients with RA and available CVD data [median symptom duration 7 months; interquartile range 5–10] were included. Characteristics of patients at baseline are shown in Table 1.

**Table 1:** Characteristics of patients at inclusion

	Patients (n = 232)
**Demographics and history**	
Female sex, n (%)	164 (71)
Age at inclusion (years)	63 (52–72)
Symptom duration at inclusion (months)	7 (5–10)
Time to first DMARD ^a^ (months)	5 (3–7)
**Traditional CVD risk factors**	
Hypertension, n (%)	66 (28)
Diabetes, n (%)	14 (6)
Hyperlipidaemia, n (%)	19 (8)
BMI (kg/m^2^)	25 (22–28)
Cigarette smoking status	
Current smokers, n (%)	78 (35)
Previous smokers, n (%)	75 (34)
Never smokers, n (%)	69 (31)

Median (IQR) given unless otherwise stated

DMARD, disease-modifying antirheumatic drugs; CVD, cardiovascular disease; BMI, body mass index; IQR, inter quartile range

Missing numbers were as follows: Time to DMARD = 22, BMI = 10, Cigarette smoking status = 10

^a^ Duration from rheumatoid arthritis symptom onset to start of first DMARD.

Disease parameters and treatment at baseline and at the 2-year follow-up are shown in Table 2.

**Table 2:** Treatment and disease parameters at inclusion and at 2 years

	Inclusion (n = 232)	2 years (n = 207) ^a^
**Current treatment**		
DMARD (any), n (%)	190 (82)	171 (83)
MTX, n (%)	123 (53)	125 (60)
MTX dose (mg/week)	10 (7.5–10.0)	10 (7.5–17.5)
Other DMARDS, n (%)	67 (29)	43 (21)
Concurrent prednisolone, n (%)	89 (38)	62 (30)
Prednisolone dose (mg/day)	7.5 (5.0–15.0)	5 (2.5-6.0)
**Disease parameters**		
RF-positive at inclusion, n (%)	143 (62)	N/A
Anti-CCP antibody-positive at inclusion, n (%)	115 (57)	N/A
Erosions present ^b^, n (%)	35 (15)	67 (32)
COMP (units/L)	11 (9–14)	N/R
DAS28	4.7 (3.6–5.7)	3.5 (2.6–4.5)
Low disease activity ^c^, n (%)	39 (17)	85 (41)
Moderate disease activity ^c^, n (%)	103 (45)	93 (45)
High disease activity ^c^, n (%)	89 (39)	27 (13)
HAQ	0.75 (0.38–1.25)	0.50 (0.00–1.00)
ESR (mm/h)	21 (10–43)	15 (8–26)
CRP (mg/l)	9 (< 9–28)	< 9 (< 9–11)

Median (IQR) given unless otherwise stated

DMARD, disease-modifying antirheumatic drugs; MTX, methotrexate; RF, rheumatoid factor; N/A, not applicable; Anti-CCP, anti-cyclic citrullinated peptide antibodies; COMP, cartilage oligomeric matrix protein; N/R, not reported; DAS28, disease activity score in 28 joints; HAQ, health assessment questionnaire; ESR, erythrocyte sedimentation rate; CRP, C-reactive protein; IQR, inter quartile range

For treatment and disease parameters at inclusion/two years, missing numbers were as follows: Anti-CCP = 31/-, Erosion present = 0/7, COMP = 30/-, DAS28 = 1/2, HAQ = 1/0, ESR = 0/2.

^a^ Patients with a clinical follow-up at 2 years

^b^ Determined by a radiologist as part of standard clinical practice

^c^ DAS28-classifications: Low ≤ 3.2; Moderate > 3.2-≤5.1; High > 5.1

A majority of the patients was treated with methotrexate (MTX), and 14% of all patients in the cohort (n = 32) were treated with a biologic DMARD at some time during the first 5 years. At inclusion, 35% were current- and 69% ever smokers. A documented history of hypertension, diabetes and hyperlipidaemia before time of RA diagnosis was found in 28%, 6% and 8% of the patients.

### Distribution of CVD after study start

There were 20 patients with a diagnosis of CVD, and 13 with CAD, before inclusion. These were excluded from the analyses of potential baseline predictors of respective outcomes. From inclusion to end of follow-up (2019), CVD occurred in 70 patients and CAD in 52. Since there were only 32 and 19 patients respectively with incident peripheral artery disease and cerebrovascular disease during follow-up, analysis of predictors of these outcomes were not considered feasible. Numbers and distribution of CVD and subcategories are shown in Supplementary table S2.

### Impact of variables at inclusion on the risk of CVD and CAD during follow-up

In both crude and adjusted analyses, increasing age and male sex were strongly associated with a higher risk of CVD and CAD (Tables 3 and 4).

**Table 3:** Impact of traditional CVD risk factors and disease characteristics at inclusion on the risk of CVD

	Crude		Adjusted ^a^	
Variables at inclusion	HR	95% CI	HR	95% CI
**Demographics and history**				
Male sex	**2.14**	**1.32–3.49**	**2.21**	**1.35–3.63**
Age (per year)	**1.08**	**1.06–1.11**	**1.08**	**1.05–1.11**
Time to DMARD (per SD) ^b^	0.81	0.56–1.18	1.05	0.71–1.55
**Traditional CVD risk factors**				
Hypertension	**2.81**	**1.75–4.52**	**1.91**	**1.17–3.11**
Diabetes	**3.74**	**1.78–7.86**	1.39	0.64–3.01
Hyperlipidaemia	1.38	0.50–3.78	0.73	0.26–2.06
BMI (per SD) ^b^	1.04	0.82–1.32	0.96	0.73–1.26
Cigarette smoking status				
Never smoking (reference)	1.00		1.00	
Current	1.41	0.78–2.53	1.65	0.85–3.19
Previous	1.33	0.73–2.40	0.95	0.50–1.83
Ever	1.35	0.81–2.27	1.23	0.71–2.14
**Baseline disease parameters**				
RF positivity	0.80	0.50–1.28	0.92	0.57–1.50
Anti-CCP positivity	0.94	0.57–1.56	1.12	0.66–1.88
COMP (per SD) ^b^	**1.45**	**1.17–1.80**	1.32	1.00-1.74
COMP categories				
Below median (reference)	1.00		1.00	
3rd quartile	**1.94**	**1.05–3.57**	1.06	0.55–2.04
4th quartile	**2.62**	**1.43–4.82**	1.72	0.92–3.22
DAS28 (per SD) ^b^	1.07	0.84–1.35	0.95	0.75–1.21
Disease activity ^c^				
Moderate/low (reference)	1.00		1.00	
High	1.37	0.85–2.20	1.11	0.67–1.85
HAQ (per SD) ^b^	1.09	0.86–1.39	0.97	0.76–1.24
ESR (per SD) ^b^	**1.30**	**1.05–1.61**	1.08	0.86–1.35
CRP below median (reference)	1.00		1.00	
CRP above median (> 9 mg/l)	**2.02**	**1.25–3.28**	1.12	0.68–1.83

Numbers in bold indicate significance (p < 0.05)

CVD, cardiovascular disease; HR, hazard ratio; CI, confidence interval; DMARD, disease-modifying antirheumatic drugs; SD, standard deviation; BMI, body mass index; RF, rheumatoid factor; Anti-CCP, anti-cyclic citrullinated peptide antibodies; COMP, cartilage oligomeric matrix protein; DAS28, disease activity score in 28 joints; HAQ, health assessment questionnaire; ESR, erythrocyte sedimentation rate; CRP, C-reactive protein

Analyses are based on 70 patients with first ever diagnosis of CVD during follow-up

^a^ Adjusted for age, sex, hypertension and diabetes at inclusion

^b^ SD: Time to DMARD 5.1 months; BMI 4.1 kg/m2; COMP 3.6; DAS28 1.4; HAQ 0.62; ESR 26 mm/h

^c^ For definitions see Table 2

**Table 4:** Impact of traditional CVD risk factors and disease characteristics at inclusion on the risk of CAD

	Crude		Adjusted ^a^	
Variables at inclusion	HR	95% CI	HR	95% CI
**Demographics and anthropometrics**				
Male sex	**2.88**	**1.67–4.98**	**2.87**	**1.65-5.00**
Age (per year)	**1.07**	**1.04–1.10**	**1.07**	**1.04–1.10**
Time to DMARD (per SD) ^b^	0.78	0.48–1.25	0.94	0.56–1.58
**Traditional CVD risk factors**				
Hypertension	**2.51**	**1.44–4.35**	1.75	0.99–3.12
Diabetes	**4.21**	**1.89–9.38**	1.64	0.70–3.81
Hyperlipidaemia	2.14	0.85–5.37	1.25	0.47–3.33
BMI (per SD) ^b^	1.02	0.77–1.34	0.93	0.67–1.28
Cigarette smoking status				
Never smoking (reference)	1.00		1.00	
Current	1.75	0.89–3.45	1.72	0.81–3.65
Previous	1.31	0.64–2.68	0.80	0.35–1.79
Ever	1.52	0.82–2.81	1.22	0.63–2.36
**Baseline disease parameters**				
RF positivity	0.66	0.38–1.13	0.77	0.44–1.34
Anti-CCP positivity	0.79	0.45–1.41	0.95	0.52–1.73
COMP (per SD) ^b^	**1.51**	**1.18–1.92**	1.35	0.98–1.85
COMP categories				
Below median (reference)	1.00		1.00	
3rd quartile	2.00	0.98–4.11	1.02	0.47–2.20
4th quartile	**2.73**	**1.36–5.46**	1.55	0.75–3.22
DAS28 (per SD) ^b^	1.05	0.80–1.38	0.95	0.72–1.25
Disease activity ^c^				
Moderate/low (reference)	1.00		1.00	
High	1.38	0.80–2.39	1.05	0.59–1.88
HAQ (per SD) ^b^	1.11	0.85–1.46	1.01	0.76–1.33
ESR (per SD) ^b^	1.19	0.93–1.54	1.00	0.76–1.30
CRP below median (reference)	1.00		1.00	
CRP above median (> 9 mg/l)	1.56	0.90–2.70	0.92	0.52–1.60

Numbers in bold indicate significance (p < 0.05)

CVD, cardiovascular disease; CAD, coronary artery disease; HR, hazard ratio; CI, confidence interval; DMARD, disease-modifying antirheumatic drugs; SD, standard deviation; BMI, body mass index; RF, rheumatoid factor; Anti-CCP, anti-cyclic citrullinated peptide antibodies; COMP, cartilage oligomeric matrix protein; DAS28, disease activity score in 28 joints; HAQ, health assessment questionnaire; ESR, erythrocyte sedimentation rate; CRP, C-reactive protein

Analyses are based on 52 patients with first ever diagnosis of CAD during follow-up

^a^ Adjusted for age, sex, hypertension and diabetes at inclusion

^b^ SD: Time to DMARD 5.1 months; BMI 4.1 kg/m2; COMP 3.6; DAS28 1.4; HAQ 0.62; ESR 26 mm/h

^c^ For definitions see Table 2

In univariate analysis, the presence of hypertension and diabetes were significant predictors of both CVD and CAD while hyperlipidaemia, BMI and smoking were not. When including age, sex, hypertension and diabetes in the multivariate models, hypertension remained significantly associated with CVD [adjusted hazard ratio (HR) 1.91; 95% confidence interval (CI) 1.17–3.11], whereas there were no independent associations with diabetes (Tables 3 and 4).

Baseline ESR and dichotomized CRP levels were significant predictors of CVD in crude models, but not in adjusted models and not for CAD. Positive associations were observed for baseline COMP analysed as a continuous variable with both CVD and CAD, although they did not reach significance in the adjusted models (p = 0.054 and p = 0.067) (Tables 3 and 4). When additionally adjusting for ESR, results were similar (HR 1.32, 95% CI 0.99–1.74 and HR 1.35, 95% CI 0.99–1.86). With COMP categorized according to quartiles with quartiles 1–2 (i.e., below the median) set as reference, significant associations were seen in the unadjusted models, with levels of the 3rd and 4th quartile being associated with an increased risk of CVD, and the 4th quartile of COMP with CAD. Neither of DAS28, HAQ, serological status or time to first DMARD start were associated with CVD or CAD.

### Distribution of CVD after the 2-year follow-up

Twenty-three patients with a diagnosis of CVD, and 13 with CAD, before the two-year visit were excluded from the analyses of potential predictors of subsequent outcomes. After the two-year visit, during the follow-up time ranging from 1997 to 2019, CVD occurred in 56 patients and CAD in 45. Since there were only 25 and 17 patients respectively with incident peripheral artery- and cerebrovascular disease, analysis of predictors of these outcomes were not considered feasible. Numbers and distribution with CVD and each subcategory are shown in Supplementary table S2.

### Impact of variables up to the 2-year follow-up on the risk of subsequent CVD and CAD

Disease activity parameters at 2 years had a significant impact on the risk of subsequent CVD as well as CAD (Tables 5 and 6).

**Table 5:** Impact of disease activity parameters at 2 years and up to 2 years on the risk of subsequent CVD

	Crude		Adjusted ^a^	
Variables at 2 years	HR	95% CI	HR	95% CI
DAS28 (per SD) ^b^	1.14	0.87–1.50	1.26	0.93–1.70
Disease activity ^c^				
Low (reference)	1.00		1.00	
Moderate	0.93	0.52–1.66	0.82	0.43–1.56
High	1.75	0.80–3.81	**2.58**	**1.10–6.04**
HAQ (per SD) ^b^	1.11	0.85–1.46	1.18	0.90–1.57
ESR (per SD) ^b^	**1.40**	**1.10–1.78**	**1.34**	**1.02–1.76**
CRP below 75th percentile (reference)	1.00		1.00	
CRP above 75th percentile (> 12 mg/l)	**2.13**	**1.23–3.69**	1.66	0.91–3.05
**Variables up to 2 years**				
DAS28 (AUC per SD) ^b^	1.17	0.90–1.54	**1.37**	**1.02–1.84**
HAQ (AUC per SD) ^b^	1.09	0.83–1.45	1.08	0.81–1.44
ESR (AUC per SD) ^b^	**1.39**	**1.10–1.77**	**1.31**	**1.02–1.69**

Numbers in bold indicate significance (p < 0.05)

CVD, cardiovascular disease; HR, hazard ratio; CI, confidence interval; DAS28, disease activity score in 28 joints; SD, standard deviation; HAQ, health assessment questionnaire; ESR, erythrocyte sedimentation rate; CRP, C-reactive protein; AUC, area under curve

Analyses are based on 56 patients with first ever diagnosis of CVD during subsequent follow-up

^a^ Adjusted for age, sex, hypertension and diabetes at inclusion

^b^ SD: DAS28 1.4; HAQ 0.65; ESR 18 mm/h; DAS28 AUC 27; HAQ AUC 13; ESR AUC 391

^c^ For definitions see Table 2

**Table 6:** Impact of disease activity parameters at 2 years and up to 2 years on the risk of subsequent CAD

	Crude		Adjusted ^a^	
Variables at 2 years	HR	95% CI	HR	95% CI
DAS28 (per SD) ^b^	1.24	0.92–1.66	**1.38**	**1.01–1.88**
Disease activity ^c^				
Low (reference)	1.00		1.00	
Moderate	1.05	0.54–2.04	1.17	0.59–2.31
High	2.23	0.98–5.05	**3.26**	**1.37–7.73**
HAQ (per SD) ^b^	1.20	0.90–1.60	1.30	0.96–1.74
ESR (per SD) ^b^	**1.54**	**1.23–1.94**	**1.48**	**1.16–1.88**
CRP below 75th percentile (reference)	1.00		1.00	
CRP above 75th percentile (> 12 mg/l)	**2.99**	**1.66–5.40**	**2.33**	**1.27–4.28**
**Variables up to 2 years**				
DAS28 (AUC per SD) ^b^	1.32	0.98–1.78	**1.51**	**1.10–2.07**
HAQ (AUC per SD) ^b^	1.19	0.90–1.59	1.21	0.91–1.62
ESR (AUC per SD) ^b^	**1.42**	**1.09–1.85**	**1.37**	**1.04–1.80**

Numbers in bold indicate significance (p < 0.05)

CAD, coronary artery disease; HR, hazard ratio; CI, confidence interval; DAS28, disease activity score in 28 joints; SD, standard deviation; HAQ, health assessment questionnaire; ESR, erythrocyte sedimentation rate; CRP, C-reactive protein; AUC, area under curve

Analyses are based on 45 patients with first ever diagnosis of CAD during subsequent follow-up

^a^ Adjusted for age, sex and hypertension at inclusion

^b^ SD: DAS28 1.4; HAQ 0.65; ESR 18 mm/h; DAS28 AUC 27; HAQ AUC 13; ESR AUC 391

^c^ For definitions see Table 2

High disease activity (defined as DAS28 > 5.1) was a strong predictor of subsequent CVD and CAD [HR 2.58; 95% CI 1.10–6.04, adjusted for sex, age, hypertension and diabetes, and HR 3.26; 95% CI 1.37–7.73, adjusted for sex, age, hypertension] respectively, compared to low disease activity (DAS28 ≤ 3.2). Moderate disease activity was not associated with an increased risk of CVD or CAD (Tables 5 and 6).

Both ESR (continuous variable) and CRP levels above 75th percentile (> 12 mg/l) at 2 years showed robust associations with subsequent CAD in crude as well as in models adjusted for age, sex and hypertension [adjusted HR per SD 1.48; 95% CI 1.16–1.88, and adjusted HR 2.33; 95% CI 1.27–4.28 respectively]. ESR predicted CVD, while for CRP a significant association was observed only in the unadjusted analysis (Table 5).

Although baseline disease activity measures showed only limited ability to predict CVD and CAD (Tables 3 and 4), cumulative ESR and DAS28 analysed as AUC from inclusion to the 2-year follow-up were significantly associated with these outcomes in the adjusted models (Tables 5 and 6).

For DAS28 at 2 years, analysed as a continuous variable, non-significant trends towards associations were observed for CVD while the association reached significance in the adjusted model for CAD.

HAQ at 2 years or its cumulative measure up to 2 years showed no significant associations with either of the outcomes.

### Relation between COMP and cumulative disease activity, and the risk of CVD during follow-up

In analyses stratified by median AUC for DAS28 over 2 years, baseline COMP was a significant predictor of CVD among those with lower cumulative disease activity [HR per SD 1.70; 95% CI 1.08–2.68], but not among those with cumulative disease activity above the median [HR per SD 1.26; 95% CI 0.90–1.76].

## Discussion

Results from this study on a cohort of patients with early RA suggests that COMP may be a novel predictor of CVD. We found persistently active disease to be associated with an increased risk of CVD as well as CAD, independent of traditional CVD risk factors.

To our knowledge, our study is the first to examine the association of COMP with CVD in RA. Our results indicate that higher baseline serum levels of COMP may be prognostic of future CVD and CAD. Interestingly, this is in direct contrast to a large study on the general population by Ueland et al. [36] where higher serum levels of COMP were associated with a lower risk of myocardial infarction (MI). This may reflect a different impact of high levels of COMP derived from increased cartilage turnover in early RA. We have in a previous study shown baseline levels of COMP to be associated with progression of joint erosions over 5 years [27], and this is in accordance with most other studies of early RA [25, 26, 37, 38]. COMP has been identified as a matrix component in atherosclerotic plaques, and it has been suggested that COMP may affect collagen fibre assembly [39] and contribute to a vulnerable plaque phenotype [24]. Interestingly, in non-RA patients with acute coronary syndromes, serum levels of COMP were positively correlated with the risk of subsequent major adverse cardiovascular events [40]. Whether a possible association of serum-COMP with CVD in RA reflects altered vascular morphology or serves as a surrogate marker for disease activity and joint damage, or both, is unknown and requires further investigation. Exploratory analyses in our study indicated that high COMP may be a risk factor for CVD among those with low disease activity. Further studies on COMP and the risk of CVD and potential underlying mechanisms are needed, preferably in larger cohorts.

Higher age, male sex, hypertension, and diabetes were significant predictors of CVD and CAD in this study. Furthermore, we found significant associations for ESR and CRP at baseline with CVD in crude but not in adjusted analyses, while no significant associations were seen for CAD. Baseline DAS28 and HAQ failed to predict CVD or CAD. Similar to our results, previous studies of early RA have shown single baseline levels of DAS28 not to be predictive of future CVD [15–17]. By contrast, DAS28 and inflammatory measures up to and including the 2-year follow-up were strongly associated with subsequent CVD and CAD. Similar to our results, others have presented associations of DAS28 over time with CVD [10, 13, 16, 18, 19], although there are some contradictory results [11, 15]. We found ESR at the 2-year follow-up, as well as its cumulative measure up to 2 years, to be significantly predictive of both outcomes, while high CRP at two years was a significant predictor of CAD. Cumulative ESR [11, 13] and CRP have been associated with CVD [11], although there are conflicting results in studies on older cohorts of early RA [15, 18]. In two large scale studies on RA with variable duration, time varying ESR [14] and CRP [41] were associated with MI.

Taken together, results from the present as well as most previous studies indicate that disease activity and inflammatory markers over time are of importance for the development of CVD, while initial disease activity and inflammation at RA onset may not be as predictive.

Since patients respond differently to anti-rheumatic treatment, high disease activity measures at RA onset does not necessarily translate into a major burden of inflammation in subsequent years of disease. It appears more important for the prevention of CVD that a substantial long-term reduction in disease activity and inflammation is achieved.

A number of studies indicate that suppression of disease activity and inflammation by DMARDs used according to modern treatment strategies has decreased the risk of CVD and related mortality in RA [42–45]. This is compatible with the concept that inflammation partly explains the excess CVD in RA.

Based on results from this study, close monitoring of patients with negative prognostic markers is suggested, and timely pharmacologic as well as non-pharmacologic interventions aimed at reducing the effects of disease activity and cardiovascular risk factors are needed to further improve CVD related outcomes in RA.

One limitation in this study was the relatively small sample size, affecting the statistical power for the multivariate analyses. Also, the patients in our cohort were included just prior to or shortly after the introduction of biologic DMARD use in RA and were classified according to the older 1987 ACR criteria. Hence, the results of this study may not apply to patients diagnosed according to more recent criteria, with more readily accessible biologics and who are treated according to a treat to target strategy [34].

Data on smoking, BMI, and COMP were only collected at baseline, therefore longitudinal assessment of the impact of these factors was not possible. Furthermore, the ICD-codes used for defining CVD in this study may not completely exclude care related to chronic CVD.

Strengths of this study includes the structured longitudinal follow-up of an inception cohort recruited from a defined catchment area. This approach minimizes the impact of selection bias, allowing for greater generalizability of our results to patients with RA seen in real-world clinical practice.

## Conclusions

Circulating COMP, which has been used as a marker of cartilage turnover in RA, may be associated with an increased risk of CVD and CAD. High levels of systemic inflammation and DAS28 at two years after diagnosis, as well as cumulative disease activity, were associated with increased risk of CVD and CAD, independent of traditional CVD risk factors. Awareness of the particularly increased CVD risk among difficult to treat patients is important in order to further improve CVD related outcomes in RA.

### Electronic supplementary material

Below is the link to the electronic supplementary material.

Supplementary Material 1

Supplementary Material 2

## Data Availability

The datasets generated and/or analysed during the current study are not publicly available due to Swedish legislation (the Personal Data Act), but a limited and fully anonymized dataset containing the individual patient data that support the main analyses is available from the corresponding author on reasonable request.

## Abbreviations

ACPA: anti-citrullinated protein antibodies
ACR: American College of Rheumatology
Anti-CCP: anti-cyclic citrullinated peptide antibodies
AUC: area under curve
BMI: body mass index
CAD: coronary artery disease
CI: confidence interval
COMP: cartilage oligomeric matrix protein
CRP: c-reactive protein
CVD: cardiovascular disease
DAS28: disease activity score in 28 joints
DMARD: disease-modifying anti-rheumatic drugs
ESR: erythrocyte sedimentation rate
EULAR: European League Against Rheumatism
HAQ: health assessment questionnaire
HR: hazard ratio
ICD: International Classification of Disease
MI: myocardial infarction
MTX: methotrexate
PH: proportional hazards
RA: rheumatoid arthritis
RF: rheumatoid factor
SD: standard deviation
WHO: World Health Organization
