# Supplementary material for: Cardiovascular disease risk in early rheumatoid arthritis: the impact of cartilage oligomeric matrix protein (COMP) and disease activity
Source: BMC Rheumatol. 2023 Dec 1;7:43. doi: 10.1186/s41927-023-00367-2 (PMC10690963; doi:10.1186/s41927-023-00367-2)
Supplement: Supplementary file 1 — Supplementary Material 1 [file 41927_2023_367_MOESM1_ESM.docx]

| **Supplementary Table S1. ICD-codes used for CVD definitions** | |
| --- | --- |
| **CVD subcategories** | ICD-codes (8-10^th^ version) |
| Coronary artery disease | 410-414 and I20-25. |
| Peripheral artery disease | 440-442, 443.90, 443.99, 443X, 444, I70-72, 173.9 and 174. |
| Cerebrovascular disease | 433-436 (excluding 433.00, 433.99 and 434A), 437A, I63-66, I670 and I672. |

ICD, international classification of diseases; CVD, cardiovascular disease.
