# Supplementary material for: Cardiovascular disease risk in early rheumatoid arthritis: the impact of cartilage oligomeric matrix protein (COMP) and disease activity
Source: BMC Rheumatol. 2023 Dec 1;7:43. doi: 10.1186/s41927-023-00367-2 (PMC10690963; doi:10.1186/s41927-023-00367-2)
Supplement: Supplementary file 2 — Supplementary Material 2 [file 41927_2023_367_MOESM2_ESM.docx]

| **Supplementary table S2. Number of patients with first ever diagnosis of CVD and respective subcategories** | | |
| --- | --- | --- |
|  | **Inclusion – End of study** | **2 years after diagnosis – End of study** |
| CVD total ^a^ | 70 (30) | 56 (24) |
| **CVD subcategories** |  |  |
| Coronary artery disease | 52 (22) | 45 (19) |
| Peripheral artery disease | 32 (14) | 25 (11) |
| Cerebrovascular disease | 19 (8) | 17 (7) |

N (%) given.

CVD, cardiovascular disease.

^a^ Total number of patients with a first ever diagnosis of coronary-, peripheral artery- or cerebrovascular disease.
